# Estimation of final standings in football competitions with a premature ending: the case of COVID-19

**DOI:** 10.1007/s10182-021-00415-7

**Published:** 2021-09-02

**Authors:** P. Gorgi, S. J. Koopman, R. Lit

**Affiliations:** 1 Vrije Universiteit, Amsterdam, The Netherlands; 2 Tinbergen Institute, Amsterdam, The Netherlands

**Keywords:** Bivariate Poisson, COVID-19, Paired-comparison models, Sport statistics

## Abstract

We study an alternative approach to determine the final league table in football competitions with a premature ending. For several countries, a premature ending of the 2019/2020 football season has occurred due to the COVID-19 pandemic. We propose a model-based method as a possible alternative to the use of the incomplete standings to determine the final table. This method measures the performance of the teams in the matches of the season that have been played and predicts the remaining non-played matches through a paired-comparison model. The main advantage of the method compared to the incomplete standings is that it takes account of the bias in the performance measure due to the schedule of the matches in a season. Therefore, the resulting ranking of the teams based on our proposed method can be regarded as more fair in this respect. A forecasting study based on historical data of seven of the main European competitions is used to validate the method. The empirical results suggest that the model-based approach produces more accurate predictions of the true final standings than those based on the incomplete standings.

## Introduction

The socio-economic impact of COVID-19 on our society has been overwhelming. Sport events have not been an exception, and they have been heavily affected by the COVID-19 pandemic. Major sport events such as the Olympic Games, the UEFA European Championship and the Tour de France have been postponed or canceled. Several ongoing sport competitions, including some of the main European football competitions, have experienced a premature ending. The premature ending of a football competition raises the issue of how to settle its final table. This has created some public debate in the media (newspapers, radio and TV) and on social media. The final standings of a competition are important to determine promotions and relegations and to select the teams that take part in international competitions, for the next season. A possible solution to determine the final standings is to consider the position of the teams in the table at the time when the competition has prematurely ended, which we refer to as the incomplete standings. This has been the mainstream approach for several football leagues. For instance, in the French Ligue 1, the average number of points per match at the time of the stop has been used to determine the final table. Similarly, in the Dutch Eredivisie, the incomplete standings has been used as the final table to determine the teams that qualify for European competitions.

The idea of using the incomplete table to determine the final standings can be justified as a ranking of the teams based on their merit in the games that have been played before the premature ending. In principle, this should reflect the expected performance in the remaining games and deliver a fair ranking of the football teams. However, the incomplete standings suffer some drawbacks for this purpose. The strength of the opposing teams in the remaining part of the competition may differ among teams. One team may have already played against all the strong teams in the competition while another team may still need to face the stronger opponents. This creates an imbalance and favors teams that have strong opponents left in the games after the premature ending. Another shortcoming of using the incomplete standings concerns home and away games. The presence of a significant home ground advantage in football matches is well documented in the literature; see, for example, Pollard (2008). Different teams can have a different number of remaining games to be played at home and this would favor teams that have already played more home games before the premature ending of the competition.

In this paper, we consider an alternative model-based approach that takes into account the strength of the opposing teams as well as the home ground advantage. We measure the performance of the teams by means of a statistical paired-comparison model. The Bradley–Terry model is a traditional example of a paired-comparison model; see Bradley and Terry (1952) and, for a review, Cattelan (2012). The outcome of a match is taken as a paired-comparison observation for the two teams that are involved in the match. In a paired-comparison model, the strength level of a team is measured relative to the strength level of the opposing team. In our analysis we determine the final ranking based on the performance of the teams in earlier match results in the season, those taking place before the premature ending. Once the strength levels of the teams have been measured for each possible match, the model can be employed to predict the expected number of points in each of the remaining games. Finally, the expected number of points at the end of the season can be used to rank the teams and obtain the final standings. We adopt a paired-comparison model that is closely related to the model of Maher (1982), which has become a standard approach in the literature to describe the outcome of football matches.

To validate whether the proposed model-based approach provides a better measure of the performance of the teams, when compared to the incomplete standings, we conduct an empirical study based on a dataset that consists of 25 seasons of competitions in seven major European football leagues (England, Spain, Germany, Italy, France, Portugal and Netherlands). For each season, we artificially stop each competition at some selected point and obtain the final table from the incomplete standings and from the model-based approach. We treat the standings that are predicted from the two methods as forecasts of the actual final table, and we measure the accuracy of the forecasts using Kendall’s tau correlation, see Kendall (1938). Finally, we construct a longitudinal test to verify whether the difference in the accuracy of the forecasts from the two methods is statistically significant. The results show evidence that the model-based standings better reflect the true final standings. These findings suggest that a model-based method is more accurate in determining the final table, in case the season has ended prematurely. The model-based method is more fair as it discounts the effect of the schedule of the matches.

The remainder of the paper is organized as follows. Section 2 discusses the details of our model-based approach to determine the final standings using a statistical analysis. Section 3 presents the testing methodology to compare the forecasting performance of the model-based approach with the incomplete standings in forecasting the true final table. Section 4 reports the empirical results. Section 5 concludes.

## A statistical method to estimate the final standings

When the incomplete standings are used to set the final ranking, it does not take into account the bias introduced by the schedule of the games and the different skill levels of the opposing teams, in the remaining part of the season (with games that will never be played due to the premature ending). For example, assume there are a few games left to the end of the season and consider two teams, team A and team B, with the same number of points. Team A has already played the strongest opponents while instead team B still needs to face some strong opposing teams in the last games. In such a case, it may be desirable to take into account that team A has shown a better performance than team B: although the two teams have the same number of points, team A has faced the stronger teams. Furthermore, team A has “easier” matches left to be played and therefore team A can be expected to collect more points. Another drawback of taking the incomplete table as the final ranking is that home and away games are not accounted for. Teams can have a different number of games to be played at home. It is well-known that the team playing at home obtains a higher likelihood of gaining more points from the game; see the discussions in Pollard (2008) and Buraimo et al. (2012).

The approach we propose is designed to take these factors into consideration. We measure the performance of the teams in the season by means of a paired-comparison model as in Maher (1982). The performance of the teams is obtained only using the outcomes of matches that have already been played in the same season. On the basis of this measured performance, we determine the final standings using the model-implied expected number of points in the remaining games. We should emphasize that our analysis is based on a paired-comparison model that has been widely used to model and predict football matches. In particular, the paired-comparison model for outcomes of football matches of Maher (1982) is adopted in many studies, including Dixon and Coles (1997), Goddard (2005), Karlis and Ntzoufras (2009), Hvattum and Arntzen (2010), Rue and Salvesen (2000) and Koopman and Lit (2015). Koopman and Lit (2019) However, the purpose of the current study is not to construct a predictive model using all the available data but to provide a fair measure of the performance of the teams in the current season to determine the final table. We provide a detailed description of the approach in the remainder of this section.

We denote the outcome of a football match between the home team *i* and the away team *j* as a pair of counts $(X_i,Y_j)$ for $i,j\in \{1,\dots ,n\}$, $i\ne j$, where $X_i$ is the number of goals scored by the home team *i*, $Y_j$ is the number of goals scored by the away team *j*, and *n* indicates the number of teams in the competition. We describe the match outcome by means of a bivariate Poisson paired-comparison model

$$\begin{aligned} (X_i,Y_j)\sim \mathcal {BP}(\lambda _{x,ij},\lambda _{y,ij}, \gamma ), \end{aligned},$$

where $\mathcal {BP}(\lambda _{x,ij},\lambda _{y,ij}, \gamma )$ denotes a bivariate Poisson distribution with intensity $\lambda _{x,ij}$ for the home team count, intensity $\lambda _{y,ij}$ for the away team count, and coefficient $\gamma \ge 0$ for the dependence between the two counts. The probability mass function (pmf) of the bivariate Poisson $\mathcal {BP}(\lambda _{x,ij},\lambda _{y,ij}, \gamma )$ is given by

1 $$\begin{aligned} {\mathbb {P}}(X_i=x, Y_j=y)=\frac{\lambda _{x,ij} \, \lambda _{y,ij}}{x! \, y! \, e^{\lambda _{x,ij}+\lambda _{y,ij}+\gamma }}\sum _{k=0}^{\min \{x,y\}}\left( {\begin{array}{c}x\\ k\end{array}}\right) \left( {\begin{array}{c}y\\ k\end{array}}\right) k!\left( \frac{\gamma }{\lambda _{x,ij}\lambda _{y,ij}}\right) ^k, \end{aligned}$$

for $x,y \in \{0,1,\dots ,\infty \}$. We refer to Johnson et al. (1997) for a review of the bivariate Poisson distribution and to Karlis and Ntzoufras (2003) for its original application to sport matches. The intensities $\lambda _{x,ij}$ and $\lambda _{y,ij}$ determine the difference in expected goals between the home and away teams. The intensities are specified as

$$\begin{aligned} \lambda _{x,ij}=\exp (\delta +\alpha _i+\beta _j), \quad \text {and} \quad \lambda _{y,ij}=\exp (\alpha _j+\beta _i), \end{aligned},$$

where $\alpha _k$ represents the attacking ability of team *k*, for $k=i,j$, $\beta _k$ represents the defending ability of team *k*, for $k=i,j$, and $\delta$ is the home ground advantage. The specification for the intensities originates from Maher (1982). It accounts for the different strength level of the teams $(\alpha _k,\beta _k)$, for $k=1,\dots ,n$, as well as the home ground advantage $\delta$ to determine the probability distribution of the match outcome.

In most applications of the bivariate Poisson model for sports data, the objective is to specify $\alpha _k$ and $\beta _k$ to best predict the outcomes of future matches. For instance, the model can be extended with other covariates that may explain the strength levels of the team. However, in our current study the objective is to determine a final ranking that reflects the performance of the teams in the matches that have been played earlier in the season. We achieve this by estimating the parameters of the model $(\alpha _1,\dots ,\alpha _n,\beta _1,\dots ,\beta _n,\delta ,\gamma )$ using the method of maximum likelihood (ML). The maximum likelihood estimates of the model parameters are obtained via the numerical maximization of the log-likelihood function. The likelihood function is available in closed form which allows for a fast estimation process despite the high-dimensional model challenges. We refer to Appendix A in Koopman and Lit (2019) for the details of maximum likelihood estimation.

The parameter estimates are only based on the data of the current season. In this way, the estimated intensities only reflect the performance of the teams in the current season. The estimation is subject to the standard restriction $\sum _{i=1}^n\alpha _i=0$ for the purpose of identification of the parameters, since only the differences between the attack and defense strengths are identified and not their overall level. Since the difference between attack and defense strengths identify the intensities, only the restriction $\sum _{i=1}^n\alpha _i=0$ is needed and the defence strengths are obtained in relation to this restriction, see for example Dixon and Coles (1997). Once the parameters have been estimated, we can obtain the expected number of points of each team in the remaining games and construct the final table by using the model-implied expected number of points at the end of the season. In particular, first we calculate the winning probability of the home team $p_h$, the winning probability of the away team $p_a$, and the probability that the match ends with a draw $p_d$ as follows

$$\begin{aligned} p_h=\sum _{y=0}^\infty \sum _{x=y+1}^\infty {\mathbb {P}}(X_i=x, Y_j=y),\\ p_a=\sum _{x=0}^\infty \sum _{y=x+1}^\infty {\mathbb {P}}(X_i=x, Y_j=y), \end{aligned}$$

and

$$\begin{aligned} p_d=\sum _{z=0}^\infty {\mathbb {P}}(X_i=z, Y_j=z), \end{aligned},$$

where the expression of the pmf is given in Eq. (1). Based on these probabilities, we calculate the expected number of points of the home and away teams. We consider the system of assigning 3 points to the winning team, 0 to the losing team, and 1 point to each of the teams if the game ends with a draw. This system is the standard in most football competitions. The expected number of points of the home team $ep_{h}$ and the one of the away team $ep_{a}$ are

$$\begin{aligned} ep_{h} = 3 p_h + p_d, \quad \text {and} \quad ep_{a} = 3 p_a + p_d. \end{aligned}$$

The final table is obtained by summing up the expected number of points of each team in the remaining games and adding these expected points to the realized points of the incomplete table.

Our proposed method relies on the ML estimates of the parameters in the paired-comparison model to obtain the final table. The uncertainty of the parameter estimates is not accounted when we determine the expected number of points of the teams. However, the method can be extended such that the derivation of the expected number of points takes into account the sampling distribution of the ML estimates. This extension can be achieved via the parametric bootstrap method or via the re-sampling of the parameters from a Gaussian approximation of the sampling distribution of the parameter estimates (typically based on their asymptotic distribution). A more detailed discussion on this adjustment can be found in Appendix A. The empirical results indicate that the expected number of points as obtained by accounting for parameter uncertainty are close to those obtained with the standard model-based ML method described above. The additional results for the final rankings of the teams are presented in Appendix A and they are almost the same overall.

The proposed model-based approach ranks the teams based on the expected number of points. Therefore, it is highly unlikely that two or more teams are tied in the same position of the table since the expected number of points is continuous, not discrete. However, it is theoretically possible for ties to occur. Typically, if two teams end the season with the same number of points, their ranking is based on goal difference (that is, goals scored minus goals conceded). If the goal difference is also equal, the team with more goals scored is then ranked higher. These rules apply to the English Premier league, for example. Apart from the expected number of points, our model-based approach can also produce the expected number of goals scored and conceded, as the model relies on the bivariate Poisson distribution. Therefore, it can be used to break ties according to rules as described above.

## Testing the relative accuracy of estimated final standings

Once the final table is estimated using the method of the previous section, we need to verify whether the model-based approach provides a better estimate of the final ranking of the teams, compared to taking the incomplete table as the final ranking. We emphasize that both the model-based estimate of the final standings and the incomplete standings are based on the same data set consisting of all match results in the season, before the premature ending of the competition. Both estimates can be interpreted as forecasts of the true final table. We can use historical data from several football competitions to test whether the model-based approach performs better than the incomplete standings in terms of forecast accuracy. Next we present our proposed testing methodology to compare the two forecasts of the final table.

We can assess the accuracy of a final table forecast using the Kendall’s tau correlation coefficient between the predicted and the actual table rankings. The Kendall’s tau statistic is a correlation measure between two rankings. The higher the correlation, the more similar the rankings. Assume that we have a historical dataset of *K* competitions observed over *T* seasons. We can select a premature stopping time for the seasons, for instance, after a certain percentage of games have been played in the competition. Then, for competition *k*, with $k=1,\dots ,K$, and season *t*, with $t=1,\dots ,T$, we obtain two Kendall’s tau correlations: (i) between the model-based prediction and the true final table $\tau _{k,t}^m$, and (ii) between the incomplete table prediction and the true final table $\tau _{k,t}^c$. We define the difference between the two correlation coefficients $\tau _{k,t}^m$ and $\tau _{k,t}^c$ as $d_{k,t}=\tau _{k,t}^m-\tau _{k,t}^c$. Next, we can formally test the hypothesis that the expected difference $\mu _k=\text {E}(d_{k,t})$ is different from zero. When $\mu _k>0$, the model-based prediction is more accurate than the incomplete table prediction in forecasting the true final table. Hence, we consider the following test hypothesis

2 $$\begin{aligned} H_0:\ \mu _k = 0 \quad \text {against} \quad H_1:\ \mu _k \ne 0. \end{aligned}$$

The test statistic is given by

$$\begin{aligned} s_k = \sqrt{T} \, \frac{{\bar{d}}_k}{\widehat{\sigma }_k}, \end{aligned}$$

where *T* is the sample size (number of seasons) and

$$\begin{aligned} {\bar{d}}_k=\frac{1}{T}\sum _{t=1}^T d_{k,t}, \qquad \widehat{\sigma } _k =\sqrt{\frac{1}{T}\sum _{t=1}^T (d_{k,t}-{\bar{d}}_k)^2}. \end{aligned}$$

Under standard conditions, the statistic $s_k$ above has an approximate standard normal distribution. We note that the standard deviation ${\hat{\sigma _k}}$ is obtained under the assumption that the difference of Kendall’s tau statistics $d_{k,t}$ is not autocorrelated over seasons *t*. This assumption may be relaxed using a robust estimate of the standard error. However, we have not found any evidence of serial autocorrelation in the series $d_{k,1},\ldots ,d_{k,T}$, for any $k=1,\ldots ,K$, in our empirical study of Sect. 4.

The hypothesis (2) can be considered to verify whether the model-based predictions are more accurate for a given competition *k*, for $k=1,\dots ,K$. The drawback of testing for each competition separately is that the test statistics will have low power to reject the null hypothesis since the number of seasons *T* is relatively low. We therefore also consider a longitudinal test to pool information from multiple competitions together. We refer to this test as longitudinal because it relies on both the time series dimension and the cross-sectional dimension, where each competition is a cross-sectional unit that is observed repeatedly over time. The longitudinal test is based on the hypothesis

3 $$\begin{aligned} H_0:\ \frac{1}{K}\sum _{k=1}^K\mu _k = 0 \quad \text {against} \quad H_1:\ \frac{1}{K}\sum _{k=1}^K\mu _k \ne 0. \end{aligned}$$

If we assume that the expected difference in the Kendall’s tau coefficients is the same for all competitions $\mu _k=\mu$, for $k=1,\dots ,K$, the test hypothesis in (3) reduces to $H_0: \mu = 0$ against $H_1: \mu \ne 0$. We retain the general form of the test without assuming $\mu _k=\mu$ to avoid unnecessary assumptions. The corresponding test statistic is given by

$$\begin{aligned} s = \sqrt{KT} \, \frac{{\bar{d}}}{\widehat{ \sigma }}, \end{aligned}$$,

where

$$\begin{aligned} {\bar{d}} =\frac{1}{K}\sum _{k=1}^K {\bar{d}}_k, \quad \widehat{ \sigma } =\sqrt{\frac{1}{K}\sum _{k=1}^K {\hat{\sigma }}_k^2}. \end{aligned}$$

Under standard regularity conditions, the test statistic *s* has an approximate standard normal distribution. We emphasize that the test remains valid, even if the variance of $d_{k,t}$, that is $\sigma ^2_k={\mathbb {V}}ar(d_{k,t})$, is different for each league. This robustness to heterogeneity in the variances is relevant since different leagues can have a different number of teams and therefore the precision (variance) of the Kendall’s tau measures may vary across leagues.

## Empirical evidence for seven European football leagues

In our empirical study, we consider seven of the main European football competitions. Two of these competitions, France and the Netherlands, are of particular interest since in these countries the 2019/2020 football season was stopped prematurely due to the COVID-19 restrictions imposed by the government. The other competitions included in the study are the football leagues of England, Spain, Germany, Italy, and Portugal. The empirical analysis is based on historical data from the season 1994/1995 to the season 2018/2019 for the group of seven countries. Hence, we have 25 seasons for each competition. All computations and analyses are done by the software package *Time Series Lab—Sports Statistics Edition* of Lit (2020) which is freely available at https://timeserieslab.com.^1^ A step-wise description of how to replicate the results in Tables 3 and 4 using the software is described in Appendix B.

### Testing the superior precision of the model-based approach

We apply the testing methodology described in Sect. 3. We consider several premature ending times of the seasons to see how the stopping point affects the results. In particular, we report the test results for the following percentages of games that have already been played before the stop of the season occurs: 50%, 60%, 70%, 80%, 90% and 95%. Table 1 reports the test statistics for each competition as well as the test statistic of the longitudinal test that includes all the competitions. If we focus on the test for each individual competition presented in Eq. (2), we can see that most of the test statistics are not significant at a confidence level of 0.05 or 0.1. This is especially the case for Germany, Spain, Portugal and the Netherlands. We note that the test results for a single competition are based on a very small sample size, $T=25$. Furthermore, small changes in the number of points of the teams can produce large variations in the Kendall’s tau correlation as the ordering of the teams can change significantly. Therefore, the test statistics are highly affected by sampling uncertainty and the power of the test is expected to be low. The strong effect of sampling uncertainty can also be noted by how the test statistic of a given competition can differ depending on the completion level. For example, the results of the English competition are positive and significant for most completion levels except at 70% where the statistic is not only insignificant but even negative. An alternative interpretation to the lack of statistical significance is that there may be some heterogeneity such that in some competitions it is less relevant to take into account the effect of the schedule. However, given the small sample size, it is difficult to make any conclusion in this respect. Overall, we see that most of the test statistics are positive. This suggests that the forecast of the end of the season standings obtained using the model-based approach may be more accurate than the incomplete table.

**Table 1:** Test statistics for the seven competitions for different completion levels of the seasons

	50%	60%	70%	80%	90%	95%
England	3.14***	2.71***	− 0.21	3.44***	2.53**	0.60
Spain	0.53	1.35	0.94	1.03	1.24	− 0.05
Germany	− 1.10	0.65	0.91	1.29	− 1.14	− 0.70
Italy	1.99**	2.57**	1.68*	1.11	1.88*	2.09**
France	− 0.01	0.04	2.00**	1.34	1.20	0.45
Portugal	− 0.96	− 1.79*	0.83	− 0.05	1.20	1.37
Netherlands	− 0.32	0.34	− 1.17	0.36	1.60	0.13
All countries	0.63	1.83*	2.05**	3.23***	3.10***	1.25

The last row reports the statistics for the longitudinal test that includes all seven competitions. The reported significance of the test is indicated by $$^{*}$$(10% level), $$^{**}$$(5% level), and $$^{***}$$(1% level)

We focus next on the results of the longitudinal test in Eq. (3) that includes all seven competitions. For this test, the actual sample size is larger since the information from the cross sectional dimension is also exploited. The total number of observations used for the test is $KT=175$, where $K=7$ and $T=25$. Therefore, this test is expected to have more power. From the results, we can see that the test statistics are positive for all the completion levels of the competitions. Furthermore, the test is significant at 1% level for 80% and 90% completion of the season, it is significant at 5% level for 70% completion of the season, and it is significant at 10% level for 60% completion of the season. Instead, the results are not significant for 50% and 95% completion of the season. These findings are not surprising. When 50% of the season is completed, the teams are facing all opponents in the remaining games of the season. Therefore, the model-based approach will tend to produce similar results as the incomplete table. Differences can be due to the fact that the model-based approach measures the skills of the teams based on goals that are scored and conceded, instead, the incomplete table only accounts for the points, irrespective of the number of goals. When the completion level of the season is 95%, we also do not expect major differences in performance between the methods. In this case, there are only two games left to be played and therefore changes in the final table are less likely to occur.

In practice, the proposed method will be applied on a case-by-case basis: for a given season and a given competition as considered in Sect. 4.2 for the Dutch and French competitions in 2019/2020. Clearly, there is no guarantee that the model-based approach is more accurate than the incomplete standings for a given season and competition because of sampling uncertainty. As discussed before, the idea of the method is to obtain a final table that better reflects the merit of the teams and removes the bias caused by the schedule of the games. In this respect, we conclude that the test results validate the model-based approach in terms of prediction accuracy as the results for the longitudinal test provide statistical evidence of a better performance compared to the incomplete table.

The results discussed so far are based on the Kendall’s tau correlation coefficient, which provides a measure of similarity between the predicted and the actual standings. In practice, the top and bottom positions of the table are more important than the middle positions of the table. The top positions determine which teams qualify to international competitions while the bottom positions determine which teams are relegated. When comparing the top and bottom of two rankings, Kendall’s tau may not be an appropriate measure of correlation since the teams in both the top and bottom positions of the two rankings can be different. As an alternative measure of closeness between the estimated and actual standings, we consider the proportion of teams that are correctly classified to be in the top and bottom of the table. Assume that we are interested in the top 3 and the bottom 3 positions. We then calculate the proportion of teams that are present in the top and bottom 3 positions, for both the estimated and the actual standings. Furthermore, assume that, apart from the actual ordering, the top three teams of the estimated standings are the same as the top 3 teams in the actual standings (also assume that the same is true for the bottom 3 teams). Then, the proportion of teams correctly classified to be in the top and bottom 3 positions of the table is equal to 1. Similarly, if only 3 out of 6 teams are correctly assigned to the top and bottom 3 of the table, the proportion equals 0.5.

The testing methodology presented in Sect. 3 can be applied to this proportion of teams that are correctly classified. We simply replace the Kendall’s tau correlation with the proportion of correctly classified teams in the top and bottom of the table. The difference in accuracy between model-based and incomplete standings can be tested accordingly. Table 2 reports the test statistics of the longitudinal test in Eq. (3) for top and bottom 2, 3, 4 and 5 positions of the table and for different completion levels. We find that the test statistics are positive in almost all cases and they are significant in some instances. This empirical finding indicates that the model-based approach tends to have an overall better performance. The relatively weaker statistical evidence in Table 2 compared to the results based on the Kendall’s tau in Table 1 may be due to the percentage of correctly classified teams in the top and bottom of the table: this percentage does not account for the ordering of the teams but only for their correct classification. Therefore, the statistic is more sensitive to sampling uncertainty and is therefore less powerful in testing the difference in performance.

**Table 2:** Test statistics of the longitudinal test for the percentage of teams correctly classified in the top and bottom of the table

Number teams	50%	60%	70%	80%	90%	95%
2	0.53	0.27	1.31	1.20	1.00	0.22
3	1.60	1.16	0.67	1.18	3.26***	0.17
4	1.55	1.38	2.93***	1.83*	1.81*	0.16
5	0.11	− 0.11	1.75*	1.41	1.56	2.04**

The first column indicates the size of the top and bottom classifications (in number of teams). The reported significance of the test is indicated by $$^{*}$$(10% level), $$^{**}$$(5% level), and $$^{***}$$(1% level)

### The premature endings of the French and Dutch competitions

Finally, we apply the method to the 2019/2020 season of the French Ligue 1 and the Dutch Eredivisie. Both these competitions were stopped and the final standings were settled using the incomplete table. For the French competition, the average point per match was used to determine the final standings. Obviously, if the teams have played the same number of games, then the average point per match and the incomplete standings give the same ranking of the teams. For the Dutch competition, the incomplete table was used to determine the final standings and select the teams entering the European competitions in the season 2020/2021.^2^ Tables 3 and 4 report the incomplete table and the model-based table for the French Ligue 1 and the Dutch Eredivisie, respectively.

We learn from Table 3 that the most relevant difference in the French rankings of the Ligue 1 teams concerns Lyon. In the model-based standings, Lyon is ranked 5th instead of 7th in the incomplete table surpassing Reims and Nice. The first 6 positions of the French Ligue 1 are important to enter the European competition and the 5th position gives access to the UEFA Europa League. The schedule of the remaining games reveal that Lyon has already played the strongest team, Paris SG. Instead, both Reims and Nice have not faced Paris SG. Furthermore, Lyon has six home matches left compared to the five of Reims and the four of Nice. This may well explain the difference between the two rankings presented in Table 3

**Table 3:** Incomplete table and model-based table of the 2019/2020 season of the French Ligue 1

	Incomplete Listings				Model-based Listings		
	Team	Points	Matches		Team	Points	Matches
1	Paris SG	68	27	1	Paris SG	94.27	38
2	Marseille	56	28	2	Marseille	71.25	38
3	Rennes	50	28	3	Rennes	66.68	38
4	Lille	49	28	4	Lille	65.16	38
5	Reims	41	28	5	Lyon	57.96	38
6	Nice	41	28	6	Reims	55.05	38
7	Lyon	40	28	7	Nice	54.36	38
8	Montpellier	40	28	8	Monaco	54.22	38
9	Monaco	40	28	9	Montpellier	53.80	38
10	Angers	39	28	10	Bordeaux	53.80	38
11	Strasbourg	38	27	11	Strasbourg	52.71	38
12	Bordeaux	37	28	12	Nantes	51.14	38
13	Nantes	37	28	13	Angers	50.72	38
14	Brest	34	28	14	Brest	45.69	38
15	Metz	34	28	15	Metz	45.13	38
16	Dijon	30	28	16	Dijon	41.40	38
17	St Etienne	30	28	17	St Etienne	40.20	38
18	Nimes	27	28	18	Nimes	36.90	38
19	Amiens	23	28	19	Amiens	33.70	38
20	Toulouse	13	28	20	Toulouse	20.50	38

In case of the Dutch Eredivisie competition, the main difference between the two rankings in Table 4 is that PSV Eindhoven is in the 3rd position in the model-based standings, instead of 4th in the incomplete standings. We notice that PSV overtakes Feyenoord in the model-based standings despite Feyenoord having played 25 games and PSV 26 before the stop of the competition occurred. When taking a closer look to the schedule of the non-played matches, a similar situation as described for the French Ligue 1 occurs. PSV has already played against Ajax, which is the strongest team in the competition, instead Feyenoord has not played Ajax. Furthermore, PSV plays five of the remaining matches at home while Feyenoord only plays four matches at home.

**Table 4:** Incomplete table and model-based table of the 2019/2020 season of the Dutch Eredivisie

	Incomplete Listings				Model-based Listings		
	Team	Points	Matches		Team	Points	Matches
1	Ajax	56	25	1	Ajax	76.99	34
2	AZ Alkmaar	56	25	2	AZ Alkmaar	75.66	34
3	Feyenoord	50	25	3	PSV	65.77	34
4	PSV	49	26	4	Feyenoord	64.94	34
5	Willem II	44	26	5	Willem II	56.96	34
6	Utrecht	41	25	6	Utrecht	54.62	34
7	Vitesse	41	26	7	Vitesse	51.66	34
8	Heracles	36	26	8	Heracles	46.33	34
9	Groningen	35	26	9	Groningen	46.20	34
10	Heerenveen	33	26	10	Heerenveen	43.51	34
11	Sparta	33	26	11	Sparta	43.39	34
12	FC Emmen	32	26	12	FC Emmen	39.58	34
13	VVV Venlo	28	26	13	Twente	37.16	34
14	Twente	27	26	14	Zwolle	37.03	34
15	Zwolle	26	26	15	VVV Venlo	33.77	34
16	For Sittard	26	26	16	For Sittard	32.02	34
17	Den Haag	19	26	17	Den Haag	27.42	34
18	Waalwijk	15	26	18	Waalwijk	20.94	34

## Conclusion

We have presented and discussed a model-based approach to determine the final standings of football competitions with a premature ending. The key advantage of the model-based approach is that it accounts for the schedule of the matches when measuring the performance of the teams and when predicting the final table. The empirical study of seven main European competitions indicates that the model-based approach tends to deliver a final table that is closer to the true final table compared to the incomplete standings. We have considered a paired-comparison model based on the bivariate Poisson distribution to measure the performance of the teams. Alternative paired-comparison models may have been employed depending on what the target performance measure is. For instance, a paired-comparison ordered probit model could be used to exclude the number of goals scored from the performance measure of the teams. However, we do not expect that our findings will be affected much by such considerations.

## Figures and Tables

**Fig. 1:**
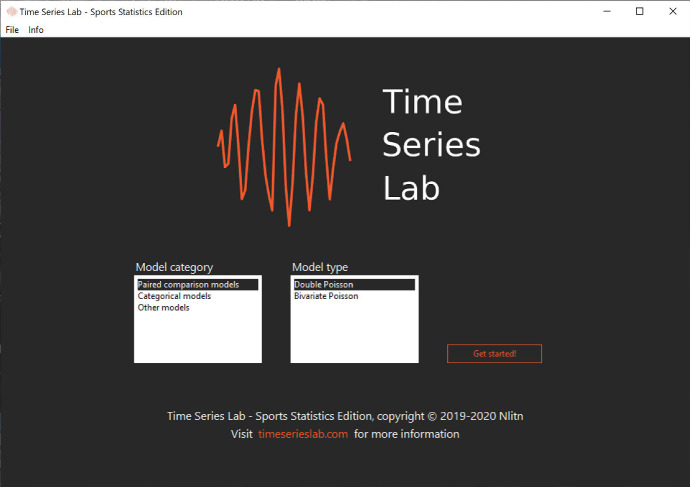
Frontpage of *Time Series Lab—Sports Statistics Edition*

**Fig. 2:**
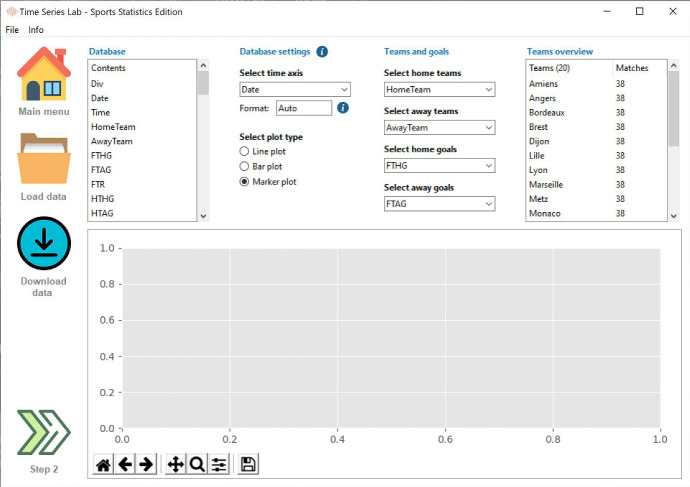
Load data page of *Time Series Lab—Sports Statistics Edition*

**Fig. 3:**
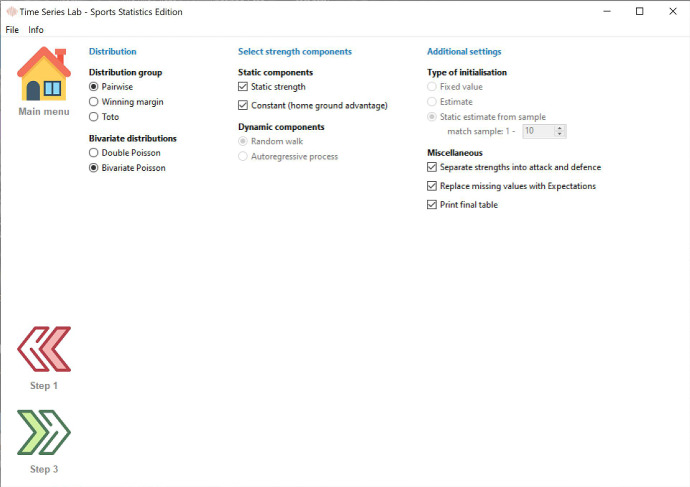
Model setup page of *Time Series Lab—Sports Statistics Edition*

**Fig. 4:**
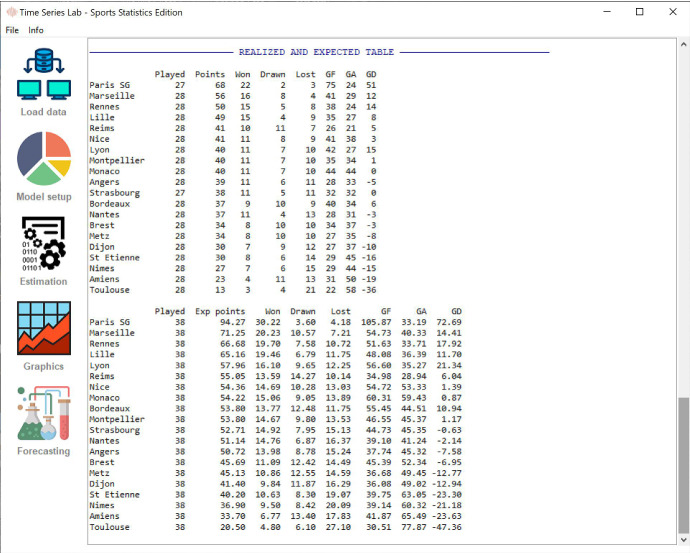
Model-based prediction of the French competition

## A Accounting for parameter uncertainty

The model-based approach presented in Sect. 2 relies on estimating the parameters of the paired-comparison model by the method of maximum likelihood (ML). Furthermore, the predictions of the expected number of points are obtained using these parameter estimates. This method does not account for parameter uncertainty of the estimates. When we consider parameter uncertainty, the prediction accuracy of the final standings may be improved as a result. Several techniques can be used for this purpose. In the context of the ML method, the uncertainty of the parameter estimates can be accounted for by using the parametric bootstrap method of Pascual et al. (2006) or by relying on the asymptotic approximation of the distribution of the estimates as in Blasques et al. (2016). For the implementation of these methods, we need to generate multiple samples from the distribution of the ML estimates and obtain a the expected number of points of the teams for each of these samples. Finally, the prediction of the final standings can obtained by averaging the expected number of points for the different samples of parameters.

We consider a simulation-based method that relies on sampling from the asymptotic distribution of the ML estimates as described in Blasques et al. (2016). The advantage of this approach is that we do not require to re-estimate the parameters of the model. We can generate parameter vectors directly from the asymptotic distribution of the parameter vector estimates. In the case of the bootstrap method, it is required to re-estimate the parameter vector from simulated data set. The re-estimation for each sample can be computationally intensive since the number of parameters is relatively large.

Table 5 reports the final tables of the 2019/2020 season for the French Ligue 1 and the Dutch Eredivisie, using the ML method that accounts for parameter uncertainty, based on 1000 parameter samples from the asymptotic distribution of the ML estimates. The reported results are very similar to those obtained from the standard ML approach that does not account for the parameter uncertainty. The differences in the expected number of points of the teams are relatively small and do not have much affect on the overall ranking of the teams compared to the standard ML approach. We find that the final rankings of the teams are the same as the ones from the model-based standings in Tables 3 and 4. In conclusion, we encounter some small differences in the expected number of points but they do not affect the ranking of the teams.

**Table 5:** Model-based table of the 2019/2020 season (using ML method accounting for parameter uncertainty) for the French Ligue 1 and the Dutch Eredivisie

	French Ligue 1				Dutch Eredivisie		
	Team	Points	Matches		Team	Points	Matches
1	Paris SG	93.92	38	1	Ajax	76.78	34
2	Marseille	71.31	38	2	AZ Alkmaar	75.48	34
3	Rennes	66.72	38	3	PSV	65.65	34
4	Lille	65.06	38	4	Feyenoord	64.78	34
5	Lyon	57.79	38	5	Willem II	56.85	34
6	Reims	55.00	38	6	Utrecht	54.58	34
7	Nice	54.34	38	7	Vitesse	51.71	34
8	Monaco	54.31	38	8	Heracles	46.34	34
9	Montpellier	53.75	38	9	Groningen	46.28	34
10	Bordeaux	53.66	38	10	Heerenveen	43.51	34
11	Strasbourg	52.82	38	11	Sparta	43.47	34
12	Nantes	51.11	38	12	FC Emmen	39.69	34
13	Angers	50.78	38	13	Twente	37.27	34
14	Brest	45.83	38	14	Zwolle	37.18	34
15	Metz	45.11	38	15	VVV Venlo	33.89	34
16	Dijon	41.55	38	16	For Sittard	32.14	34
17	St Etienne	40.26	38	17	Den Haag	27.48	34
18	Nimes	36.99	38	18	Waalwijk	21.13	34
19	Amiens	33.89	38				
20	Toulouse	20.96	38				

The results are obtained using 1000 parameter samples from the asymptotic distribution of the ML estimates

## B Software

We describe the necessary steps to replicate Tables 3 and 4 with the use of the software package *Time Series Lab—Sports Statistics Edition*, hereafter *TSL - SE*. We show how to calculate the model-based prediction of the final standings for the French competition in some simple steps. The model-based construction of the final standings for the Dutch competition is carried out in an analogous manner.

### B.1 Installing and starting

The *TSL - SE* software can be downloaded for free from https://timeserieslab.com. After installing, *TSL - SE* can be started by double-clicking the icon on the desktop or by clicking the Windows *Start* button and selecting *TSL - SE* from the list of installed programs. The frontpage of *TSL - SE*, which is visible right after the program starts, is shown in Fig. 1.

### Loading data

After pressing the *Get started* button on the frontpage you will be taken to the *Load data* step in *TSL - SE*. Click *Load data* and a file selection window opens up. Navigate to the data folder which is located in the same folder where *TSL - SE* is installed. Ctrl-click the files *F11920.csv* and *F11920_remaining.csv* so that both files are highlighted, followed by clicking the *open* button. Alternatively, the data can be downloaded from the Research section of https://timeserieslab.com. Once the data is loaded, the screen similar to Fig. 2 should appear. An indication that the correct dataset is loaded is given in the upper right corner of the screen. It shows the number of matches per team and the total number of teams in the competition. For the French competition, these are 38 and 20, respectively. Since not all scheduled matches were played in the 2019–2020 season, many missing values are part of the dataset.

### Model selection and estimation

Click the *Step 2* button which leads to the *Model setup* page. Select the Bivariate Poisson distribution and tick the boxes in front of *Replace missing values with Expectations* and *Print final table*. A screenshot of the mandatory selections is given in Fig. 3

Click the *Step 3* button which leads to the *Estimate* page. Click *Estimate* to start model estimation. After the process of maximizing the likelihood function is completed, output is printed to the Main page of the program. The model-based prediction of the final standings in the French competition is printed on screen as in Fig. 4. This printed output matches the results presented in Table 3.

## Footnotes

1 All computations and analyses are done by the software package *Time Series Lab—Sports Statistics Edition* of Lit (2020): which is freely available and can be downloaded from https://timeserieslab.com/.

2 The discussions on the premature endings of the 2019/2020 competitions in France and the Netherlands have been widely reported in the media and on social media. Although our study is not taking any position in these discussions, it is interesting that comments in the French newspaper *L’Équipe* made by the president of Lille, Gérard Lopez, can be regarded as relevant to the results presented in Table 3: https://www.lequipe.fr/Football/Actualites/Ligue-1-nous-avons-verifie-les-affirmations-de-gerard-lopez-le-president-de-lille/1130690 https://www.getfootballnewsfrance.com/2020/gerard-lopez-calls-for-end-of-2019-20-season-to-be-simulated-for-the-sake-of-fairness/
